# Causes of Time Trends in Prevalence and Mortality of Major Cancers Among U.S. Older Adults

**DOI:** 10.1093/geroni/igaa057.476

**Published:** 2020-12-16

**Authors:** Igor Akushevich, Arseniy Yashkin, Julia Kravchenko, Anatoliy Yashin

**Affiliations:** Duke University, Durham, North Carolina, United States

## Abstract

The time trend of prevalence and mortality of major cancers is the result of three competing processes: changes in the incidence rate, stage-specific survival, and ascertainment at early stages. Partitioning approach allows for evaluating the relative contribution of each of these competing processes to the overall trend. In this report we applied the partitioning methodology developed for the SEER registry data for prostate, colorectal, lung, female breast, bladder, ovarian, stomach, pancreas, kidney, liver cancers and melanoma. The analysis involves the design and estimation of four models for each cancer site: i) incidence rate using the Armitage-Doll model with individual predisposition modeled by the gamma distribution, ii) probability of relative survival after cancer diagnosis using the Weibull model for time after disease onset, iii) frequencies of stage at onset, and iv) mortality in the general population using the Gompertz model. B-splines are used to fit the time patterns of model parameters obtained for each year Relative contributions of the partitioning components were evaluated for individual cancers (e.g., increase of prevalence in prostate cancer in 2000 was due to increased incidence (59%), improved survival (29%), and improve stage ascertainment (12%)) and compared among all considered cancers. The results were discussed in the light of the effect of the accumulation of survivors occurring in early years (due to improving survival) and their higher mortality (because of higher prevalence of survivors) in later years (i.e., mortality is transferred to latter time periods due to overall improvements in survival).

